# Metabolic Syndrome (MetS), Systemic Inflammatory Response Syndrome (SIRS), and Frailty: Is There any Room for Good Outcome in the Elderly Undergoing Emergency Surgery?

**DOI:** 10.3389/fsurg.2022.870082

**Published:** 2022-06-15

**Authors:** Pietro Fransvea, Gianluca Costa, Luca Lepre, Gabriella Teresa Capolupo, Filippo Carannante, Caterina Puccioni, Alessandro Costa, Antonio La Greca, Francesco Giovinazzo, Gabriele Sganga, on behalf of the IGo-GIPS study group

**Affiliations:** ^1^ Emergency Surgery and Trauma, Fondazione Policlinico Universitario “A. Gemelli” IRCCS, Rome, Italy; ^2^ Catholic University of Sacred Heart, Rome, Italy; ^3^ Surgery Center, Colorectal Surgery Unit – Fondazione Policlinico Campus Bio-Medico, University Hospital of University Campus Bio-Medico of Rome, Rome, Italy; ^4^ General Surgery Unit, Santo Spirito in Sassia Hospital, Rome, Italy; ^5^ UniCamillus School of Medicine, -Saint Camillus International University of Health and Medical Sciences, Rome, Italy; ^6^ General Surgery and Liver Transplantation, Fondazione Policlinico Universitario A. Gemelli IRCCS, Rome, Italy; ^7^ Appendix list of Italian Group for Gastro-Intestinal Surgery Postoperative Surveillance (IGo-GIPS) Study Group Collaborators

**Keywords:** metabolic syndrome, frailty, acute care surgery, elderly, SIRS

## Abstract

**Background:**

Patients with MetS or SIRS experience higher rates of mortality and morbidity, across both cardiac and noncardiac surgery. Frailty assessment has acquired increasing importance in recent years as it predisposes elderly patients to a worse outcome. The aim of our study was to investigate the influence of MetS, SIRS, and with or without frailty on elderly patients undergoing emergency surgical procedures.

**Methods:**

We analyzed data of all patients with nonmalignant diseases requiring an emergency surgical procedure from January 2017 to December 2020. The occurrence of MetS was identified using modified definition criteria used by the NCEP-ATP III Expert Panel: obesity, hypertension, diabetes, or if medication for high triglycerides or for low HDL cholesterol was taken. Systemic inflammatory response syndrome (SIRS) was evaluated according to the original consensus study (Sepsis-1). The frailty profile was investigated by the 5-modified Frailty Index (5-mFI) and the Emergency Surgery Frailty Index (EmSFI). Postoperative complications have been reported and categorized according to the Clavien–Dindo (C–D) classification system. Morbidity and mortality have been mainly considered as the 30-day standard period definition.

**Results:**

Of the 2,318 patients included in this study, 1,010 (43.6%) fulfilled the criteria for MetS (MetsG group). Both 5-Items score and EmsFI showed greater fragility in patients with MetS. All patients with MetS showed more frequently a CACI index greater than 6. The occurrence of SIRS was higher in MetSG. LOS was longer in patients with MetS (MetSG 11.4 ± 12 days vs. *n*-MetSG 10.5 ± 10.2 days, *p* = 0.046). MetSG has a significantly higher rate of morbidity (353 (35.%) vs. 385 (29.4%), *p* = 0.005). The mortality rate in patients with MetS (98/1010, 10%) was similar to that in patients without it (129/1308, 10%). Considering patients with MetS who developed SIRS and those who had frailty or both, the occurrence of these conditions was associated with a higher rate of morbidity and mortality.

**Conclusion:**

Impact of MetS and SIRS on elderly surgical patient outcomes has yet to be fully elucidated. The present study showed a 43.6% incidence of MetS in the elderly population. In conclusion, age per se should be not considered anymore as the main variable to estimate patient outcomes, while MetS and Frailty should have always a pivotal role.

## Introduction

Although advances in surgical techniques, anaesthetic procedures, and postoperative care have all made surgery less hazardous, surgeons are generally more reluctant to operate on elderly patients because they are perceived to be frail, to have less physiological reserve, and to have more underlying medical conditions (1–3). Several factors are thought to relate to the postoperative outcome (4, 5). Metabolic syndrome (MetS) is a combination of risk factors that include high blood pressure, dyslipidaemia (high triglyceride and low high-density lipoprotein–cholesterol concentrations), high fasting glucose concentration, and central obesity (6–9). The concept of MetS, originally introduced by Dr. Gerald M Reaven as syndrome X, has evolved in the past several decades (10). Therefore, several definitions and varying criteria for MetS have been proposed (11–14). Similarly, Systemic Inflammatory Response Syndrome (SIRS) is a clinically defined state that represents activation of inflammatory, innate immune, coagulation, and repair pathways and is frequently observed in hospitalized patients. SIRS has a precise clinical definition, which has been validated in large patient populations (15–17). In this context, it has been shown by several studies that patients with MetS and/or SIRS experience higher rates of morbidity, increased instances of postoperative morbidity including cardiovascular complications, and slower recovery of function across both cardiac and non-cardiac surgery (18–20). Last but not least, frailty assessment has acquired increasing importance in recent years and it has been demonstrated that this vulnerable profile predisposes elderly patients to a worse outcome after surgery (21, 22). The aim of our study was to investigate the influence of MetS with or without SIRS, and with or without frailty on elderly patients undergoing emergency surgical procedures.

## Material and Methods

### Study Settings and Protocol

This research originates from a previous well-consolidated experience (the ERASO collaborative study group) which has led to the FRAILESEL project and related reports (23). Following a similar methodology, a new collaborative research group was founded. The IGo-GIPS (Italian Group for Gastro-Intestinal Postoperative Surveillance) is a large, nationwide network created with the aim to undertake both prospective and/or retrospective studies investigating the perioperative outcomes of specific topics mainly concerning gastrointestinal surgery. Centers were included on a volunteer basis, and neither investigators nor participating hospitals were paid for their collaboration. Clinical decisions, including operative technique, were always based on the criteria of individual centers and staff surgeons or on specific guidelines in case of intestinal obstruction (24). Although procedures were not standardized per a study protocol, it is important to note that they were likely similar among participating hospitals, with some slight technical differences across institutions seldom taken into account because they were judged to not influence the outcome. The investigators were informed about the objectives of the project and asked for complete details about the surgical management of patients following standard methods and collection protocols as already described. Data regarding patients were prospectively collected from the FRAILESEL study participating centers from January 2017 to June 2018, while data regarding other patients were retrospectively retrieved from hospital electronic databanks. The FRAILESEL Study protocol was approved by the Ethics Committee of Sapienza University and of all the centers, while no formal approval was requested for any other retrospective un-interventional study except in case of specific indication deemed by a single center. However, signed consent for the treatment and the analysis of data for the scientific purpose was obtained from all patients before any surgical procedures. This study was conducted in accordance with the Declaration of Helsinki and its later amendments. All parts of the studies and the present manuscript have been checked and presented according to the checklist for Strengthening the Reporting of Observational Studies in Epidemiology (STROBE) (25).

### Exclusion Criteria and Collected Data Confirmation

Exclusion criteria were the following: age <65 years; lack of informed consent for the study participation, if requested; patients participating in other randomized or interventional clinical trial; with regard to emergency surgery, patients were excluded if already hospitalized and scheduled for the same procedure. Sole emergency endoscopic procedures or reoperations after elective surgery were excluded. Submissions made by unconfirmed participants, duplicate submissions, records with more than 5% of missing data, and data submitted by residents from dual or more residency programs were excluded. Although demographic information was collected on the patients, all data were anonymized before analysis even for center identification.

### Patients’ Characteristics, Preoperative Variables and Objectives of This Study

For the aim of the present study, we analyzed data of all patients with nonmalignant diseases requiring an emergency surgical procedure from January 2017 to December 2020. Cancer patients were excluded because of the possible concomitant role of the neoplasm in determining an inflammatory response and/or an adverse outcome. Data collected included patient demographic characteristics (age, gender, weight, height), medical and surgical history (comorbidities), common preoperative biochemical blood examination (including PCR, PCT, and arterial blood gas analysis), pathological features, and operative details. Comorbidity was recorded if the condition was being medically treated at the time of admission, or if previous treatment for the condition was described in the admission report. The age-adjusted Charlson comorbidity index (age-CACI) was calculated and a score ≥6 was used to categorize patients having a severe comorbid condition (26, 27). The preoperative risk was assessed by the anesthesiologist-assigned American Society of Anesthesiologists (ASA). Because serum triglycerides and cholesterol were not included in blood examination at admission and waist circumference was not recorded in the FRAILESEL database, the occurrence of MetS was identified using modified definition criteria used by the NCEP-ATP III Expert Panel: (1) obesity (BMI ≥30), (2) hypertension, (3) diabetes, or (4) if medication for high triglyceride or for low HDL cholesterol was taken (28). Diagnosis of MetS required at least the presence of two of the above findings. With regard to BMI, patients were also classified in four standard categories according to the US National Heart, Lung, and Blood Institute (29). Briefly, the following terminology has been used: underweight (BMI of <18.5), normal weight (BMI of 18.5 to <25), overweight (BMI of 25 to <30), and obesity (BMI of ≥30). Systemic inflammatory response syndrome (SIRS) was evaluated according to the original consensus study (Sepsis-1) (30). SIRS criteria ≥2 met the definition of SIRS. The frailty profile was investigated by the 5-modified Frailty Index (5-mFI) and the Emergency Surgery Frailty Index (EmSFI). According to other literature reports, a 5-mFI value ≥0.4 and an EmSFI value ≥4 were adopted as a cut-off to define a patient as frailty (31, 32), but when performing statistical analysis, to simplify and make the results comparable with literature, only the 5-mFI ≥0.4 score was used for considering the frailty positive population. Postoperative complications have been reported and categorized according to the Clavien–Dindo (C–D) classification system by the study leader in each of the participating centers (33). In the statistical comparison we excluded C-D 1 grade. Morbidity and mortality have been mainly considered as the 30-day standard period definition. However, adverse outcomes have been reported regardless of the time elapsed from the surgical procedure if reasonably related to it and occurred during the hospitalization following the main emergency procedure. The cut-off adopted for CACI, 5-mFI, and EmSFI, and the choice to consider C-D complication ≥2 derives from both literature and our previous already published statistical analysis (34). The primary aim was to critically appraise the influence of MetS with or without SIRS, and with or without frailty in the elderly undergoing emergency surgical procedure.

### Statistical Analysis

Statistical analysis was carried out using IBM Corp. Released 2019, IBM SPSS Statistics for Windows, Version 26.0. Armonk, NY: IBM Corp. Dichotomous data and counts were presented in frequencies, whereas continuous data were presented as mean values ± standard deviations (SD) and/or median with 25–75 Interquartile Range (IQR), or minimum-maximum range. Differences between means were compared using the independent sample Student’s t-test or the Mann–Whitney U test when indicated. Fisher’s exact test or *χ*^2^ test, with or without Yates correction, was used to compare differences in frequencies. The role of MetS stratified for the BMI class, and the presence of SIRS and/or frailty was also assessed using the estimated adjusted odds ratio. All tests were two-tailed, and a *p*-value ≤0.05 was considered statistically significant.

## Results

### Demographics

A total of 2,318 patients fulfilling the inclusion criteria were evaluated. Table 1 reports the organ site of the procedure. The overall mean age was 77.7 ± 7.7 (range, 65–100 years), *n* = 1,175 (50.7%) were male. The overall BMI was 25.7 ± 4.5 (range, 13.3–61.2). Of the total patients included in this study, *n* = 1,010 (43.6%) fulfilled the criteria for MetS (MetS Group). Of these, *n* = 414 (38.1%) were under weight, *n* = 22 (2.0%) were normal weight, *n* = 376 (34.6%) over weight, and *n* = 275 (25.3%) obese. A 5-mFI ≥0.4 was observed in *n* = 1,203 (52.0%) patients and an EmsFI ≥4 in *n* = 887 (38.0%). A CACI >6 was observed in *n* = 870 (37.5%) patients (Table 2). The mean length of hospital stay was 10.9 ± 10.7 days and SIRS occurred in 720 (31.1%) patients. The overall morbidity rate was 31.8% (*n* = 738 pts) and a Clavien–Dindo ≥2 was present in 505 (22%) patients. (Table 2). Data about demographics, clinical features and patient’s frailty stratified by the presence of MetS are reported in Table 2.

**Table 1:** Organ site of procedure.

	*N*	%
Colon	609	26.27
Gallbladder	560	24.16
Abdominal wall	381	16.44
Adesive Small Bowel Ostruction	220	9.49
Small Bowel Ischemia	173	7.46
Appendix	138	5.95
Stomach and duodenum	128	5.52
Various digestive tract	37	1.60
Miscellaneous	24	1.04
Spleen	15	0.65
Trauma	12	0.52
Peripheral vascular system	6	0.26
Pancreas	5	0.22
Female reproductive tract	5	0.22
Esophagus	3	0.13
Liver	2	0.09
Total	2,318	100.00

**Table 2:** Demographics and clinical data stratified by the presence of MetS with or without SIRS and Frailty.

Variables	Overall 2,318 (%)	MetSG 1,010 (%)	*n*-MetSG 1,308 (%)	*p*-value
Age, y, mean ± ds	77.7 ± 7.7	78.0 ± 7.6	77.6 ± 7.8	0.298
BMI, kg/m^2^,	25.7 ± 4.5	27.3 ± 5.1	24.5 ± 3.5	<0.001
Sex, *n* (%)				0.355
Male	1,175 (50.7)	523 (4.5)	652 (55.5)	
Female	1,143 (49.3)	487 (42.6)	656 (57.4)	
5-mFI ≥ 0.4	1,203 (52.0)	744 (73.7)	459 (35.1)	<0.001
EmsFI				<0.001
EmsFI 1–3	1,435 (62.0)	573 (40.0)	862 (60.0)	
EmsFI 4–7	822 (35.5)	401 (48.8)	421(51.2)	
EmsFI 8–14	61 (2.5)	36 (59.0)	25 (41.0)	
CACI >6	870 (37.5)	451 (44.6)	419 (32.0)	<0.001
SIRS				0.001
Present	720 (31.1)	349 (34.5)	371 (28.4)	
Absent	1,598 (69.0)	661 (65.4)	937 (71.6)	
Length of stay, d,	10.9 ± 10.7	11.4 ± 11.2	10.5 ± 10.2	0.046
Morbidity overall (%)	738 (31.8)	353 (35.0)	385 (29.4)	0.005
Clavien–Dindo ≥2	505 (22.0)	245 (24.3)	260 (20.0)	0.011
Mortality (%)	227 (9.8)	98 (9.7)	129 (9.9)	0.898

### MetS Group vs *n*-MetS Group

No difference in terms of age and sex were identified between the two groups. Regarding frailty, both 5-mFI score and EmsFI showed greater fragility in patients with MetS. A 5-mFI score ≥0.4 was more frequent in the MetS Group [*n* = 744 pts (73.7%)] vs. the *n*-MetS Group [*n* = 459 pts (35.1%)] (*p* < 0.001); an EmSFI ≥ 4 was detected more frequently in MetS Group [*n* = 437 pts (43.2%)] vs. *n*-MetS Group [*n* = 446 pts (34.1%)] (*p* < 0.001). Similarly, all patients with MetS showed more frequently a CACI index ≥6 (MetS Group *n* = 451 pts (44.6%) vs *n*-MetS Group *n* = 419 (32.0%) *p* < 0.001). The occurrence of SIRS was higher in the MetS Group [*n* = 349 (34.5%) vs *n* = 371 (28.4%)] with a statistically significant difference (*p* = 0.001). We then evaluated the surgical outcomes according to the presence or absence of MetS (Table 2). LOS was longer in patients with MetS (MetS Group 11.4 ± 12 days vs. *n*-MetS Group 10.5 ± 10.2 days *p* = 0.046).

### Morbidity and Mortality

**Figure 1:**
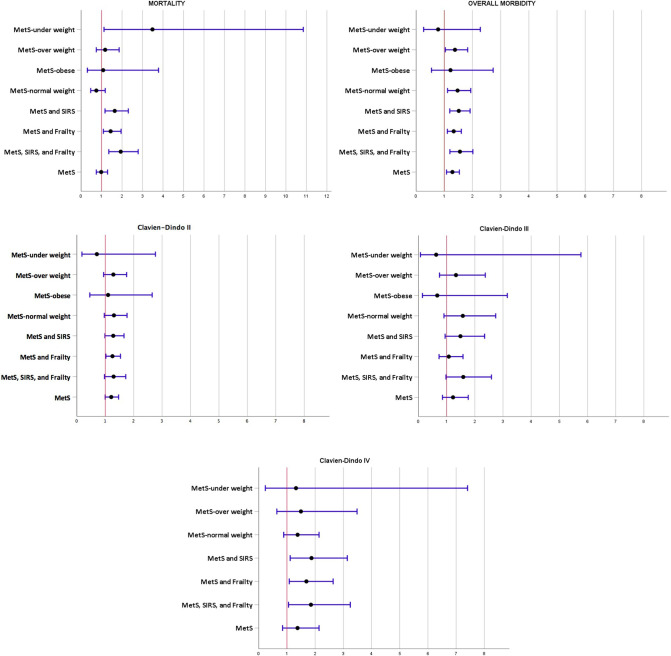
Bars showing the adjusted OR on 30-day mortality and morbidity stratified for BMI class, SIRS, and frailty. The error bars represent 95% confidence intervals (CI).

Patients with MetS have a significantly higher rate of morbidity (*n* = 353 (35.0%) vs. *n* = 385 (29.4%), *p* = 0.005). In particular, CD ≥2 was detected in *n* = 245 (24.3%) patients with MetS and in *n* = 260 (20.0%) patients without it, being the difference statistically significant (*p* = 0.011). We then conducted a sub-analysis considering separately the morbidity risk according to the Clavien–Dindo grading classification showing no association with the presence of MetS, SIRS, or frailty (Table 3). The overall mortality rate was 9.8% (*n* = 227 patients). The mortality rate in patients with MetS was similar to the mortality rate in patients without it (Table 2). Considering patients with MetS that developed SIRS and those that had Frailty or both, we found that the occurrence of these conditions was associated with a higher rate of morbidity and mortality (Table 4). The stratified estimated adjusted odds ratio analysis is reported in Table 5 and is drawn in Figure 1. Patients with MetS in which SIRS occurred and with frailty were at increased risk of mortality (SIRS odds ratio [OR] 1.643; 95% CI 1.170 to 2.308; *p* = 0.003; frailty odds ratio [OR] 1.443; 95% CI 1.088 to 1,953; *p* = 0.010; SIRS + frailty odds ratio [OR] 1.936; 95% CI 1.354 to 2.788; *p* < 0.001). Regarding the overall morbidity (CD I-IV), patients with MetS and normal weight or overweight were at increased risk (normal weight odds ratio [OR] 1.472; 95% CI 1.117 to 1,940; *p* = 0.005; overweight odds ratio [OR] 1.379; 95% CI 1.038 to 1.831; *p* = 0.025). Again, patients with MetS in which SIRS occurred and with frailty were at increased risk of overall morbidity (SIRS odds ratio [OR] 1.514; 95% CI 1.196 to 1,916; *p* < 0.001; frailty odds ratio [OR] 1.334; 95% CI 1.109 to 1.605; *p* = 0.002; SIRS + frailty odds ratio [OR] 1.555; 95% CI 1.200 to 2.015; *p *< 0.001).

**Table 3:** Comparison of overall morbidity, major complications, and mortality among patients with MetS, SIRS, and frailty

	Overall Morbidity, *n*. (%)	*p*-value	Clavien–Dindo ≥2, *n*. (%)	*p-*value	Mortality, *n*. (%)	*p*-value
MetS + SIRS (*n*.)						
Yes (349)	139 (39.8)	0.001	100 (29.0)	0.001	49 (4.0)	0.004
No (1969)	599 (30.4)		405 (20.6)		178 (9.0)	
MetS + Frailty (*n*.)						
Yes (744)	269 (36.2)	0.002	183 (25.0)	0.024	90 (12.1)	0.010
No (1574)	469 (30.0)		322 (24.5)		137 (9.0)	
MetS + SIRS + Frailty (*n*.)						
Yes (275)	112 (41.0)	0.001	80 (26.1)	0.002	44 (16.0)	<0.001
No (2043)	626 (31.0)		425 (21.0)		183 (9.0)	

**Table 4:** Comparison of Clavien–Dindo complications and MetS, SIRS, and Frailty.

	C-D I	C-D II	C-D III	C-D IV	*p*-value
MetS					
yes	108 (30.6)	144 (40.8)	59 (16.7)	42 (11.9)	0.898
no	125 (32.5)	157 (40.8)	63 (16.4)	40 (10.4)	
+SIRS present					
yes	*39* (*28.1)*	*55* (*39.6)*	*25* (*18.0)*	*20* (*14.4)*	*0*.*456*
no	*194* (*32.4)*	*246* (*41.1)*	*97* (*16.2)*	*62* (*10.4)*	* *
+Frailty present					
yes	*86* (*32.0)*	*106* (*39.4)*	*41* (*15.2)*	*36* (*13.4)*	*0*.*456*
no	*147* (*31.3)*	*195* (*41.6)*	*81* (*17.3)*	*46* (*9.8)*	* *
+ SIRS + Frailty present					
yes	*32* (*28.6)*	*43* (*38.4)*	*21* (*18.7)*	*16* (*14.3)*	*0*.*541*
no	*201* (*32.1)*	*258* (*41.2)*	*101* (*16.1)*	*66* (*10.5)*	* *

**Table 5:** Analysis of the impact of MetS on morbidity and mortality adjusted for BMI class, SIRS, and Frailty.

	OR	95% CI	*p*-value
Mortality			
MetS	0.982	0.744–1.294	0.898
Underweight	3.485	1.120–10.847	0.021
Normal Weight	0.745	0.471–1.181	0.209
Overweight	1.176	0.744–1.858	0.486
Obese	1.079	0.308–3.781	0.905
SIRS	1.643	1.170–2.308	0.003
Frailty	1.443	1.088–1.953	0.010
SIRS + Frailty	1.936	1.354–2.788	<0.001
Overall Morbidity (CDI-IV)			
MetS	1.288	1.080–1.536	0.004
Underweight	0.782	0.268–2.279	0.652
Normal Weight	1.472	1.117–1.940	0.005
Overweight	1.379	1.038–1.831	0.025
Obese	1.221	0.546–2.734	0.625
SIRS	1.514	1.196–1.916	<0.001
Frailty	1.334	1.109–1.605	0.002
SIRS + Frailty	1.555	1.200–2.015	<0.001
CD II			
MetS	1.210	0.996–1.469	0.054
Underweight	0.704	0.179–2.765	0.613
Normal Weight	1.305	0.965–1.766	0.832
Overweight	1.285	0.942–1.752	0.112
Obese	1.099	0.456–2.651	0.832
SIRS	1.281	0.988–1.660	0.060
Frailty	1.253	1.022–1.536	0.029
SIRS + Frailty	1.294	0.973–1,721	0.075
CD III			
MetS	1.226	0.851–1.766	0.273
Underweight	0.628	0.069–5.767	0.678
Normal Weight	1.574	0.904–2.742	0.678
Overweight	1.331	0.747–2.371	0.330
Obese	0.666	0.141–3.153	0.606
SIRS	1.489	0.944–2.348	0.084
Frailty	1.075	0.730–1.581	0.713
SIRS + Frailty	1.590	0.975–2.591	0.060
CD IV			
MetS	1.375	0.884–2.138	0.155
Underweight	1.320	0.232–7.406	0.751
Normal Weight	1.377	0.884–2.138	0.155
Overweight	1.495	0.641–3.486	0.342
Obese	–	–	–
SIRS	1.870	1.114–3.139	0.016
Frailty	1.689	1.081–2.637	0.019
IRS + Frailty	1.850	1.055–3.246	0.029

## Discussion

Individuals with MetS typically display symptoms of hypertension, increased fasting glucose, elevated triglycerides, obesity (either using BMI or waist circumference), and decreased high-density lipoprotein concentrations (28). The presence of any three out of five of these symptoms or risk factors constitutes a diagnosis of MetS. Using these definitions, an estimated 35–40% of the population in developed countries have MetS. Although MetS has been extensively studied in the medical arena, research about its impact on surgical patients is limited (35). In addition, the specific impact of MetS and SIRS on elderly surgical patient outcomes has yet to be fully elucidated. Based on the FRAILESEL database, the present study showed a 43.6% incidence of MetS in the elderly population with the acute surgical condition (23). This rate is higher than the 10.3% cited by Edelstein and colleagues’ review of 107,117 patients undergoing total hip and/or knee arthroplasty and the 7.9% reported by Cichos and colleagues in their study on 3,348,207 hip fracture patients, the 2.2% reported by Tracy et all in 4,489 trauma patients, the 18.1% reported by Mikolasevic et al in a cohort of 609 with acute pancreatitis and the 6.7% reported by Glance et al. in a cohort of 310,208 patients undergoing general, vascular, or orthopaedic surgery (36–40). The higher rate of MetS in our cohort is potentially explained by the demographic profile of our study population. Moreover, elderly patients with MetS are more fragile, as is underlined by the increased values of both frailty indices taken into consideration by our study. This well correlates with the evidence of a higher pre-operative CACI index. More importantly, our data showed that MetS has a significant impact on outcomes of elderly patients who underwent emergency surgical procedures. In a study of 310,208 patients with MetS undergoing noncardiac surgery, a twofold increased risk of death was observed when compared with patients without MetS (40). Bhayani and colleagues, in their research on MetS and liver resections, found a fivefold increase in myocardial infarction, a twofold increase in pulmonary complications, and a 70% increase in surgical site infections in patients with the syndrome (41). A recent meta-analysis shows that the MetS is associated with a 35% increase in the risk of all-cause mortality, a 50% increase in the risk of cardiovascular disease, and a 75% increase in the risk of stroke (42). Patients with the MetS also have a 2.6-fold increased risk of chronic kidney disease and are more likely to have impaired lung function (43, 44). Our study showed a higher incidence of SIRS in the MetSG regardless of the underlying acute surgical disease but a lower rate of positive qSOFA score. We also noted significantly higher rates of postoperative morbidity in particular CD ≥ 2 with varied complications reported across a range of surgical procedures and also a protracted length of stay. However, no statistically significant difference in the length of stay between patients with or without MetS has also been reported in several studies (40, 41, 45, 46). In literature, in addition to increased risks of morbidity, patients with MetS have a statistically significant higher risk of mortality (40, 47). However, we found that the overall mortality rate in patients with MetS was similar to the mortality rate in patients without, but considering patients with MetS that developed SIRS and those that had Frailty or both, we found that the occurrence of mortality was significantly higher. Several studies have examined the independent impact of obesity on surgical mortality after noncardiac surgery. Yet, most have failed to show that obesity is associated with increased morbidity after noncardiac surgery (48, 49). The largest study to date, by Mullen et al., also based on the ACS NSQIP database, showed a mild protective effect of BMI on mortality for overweight and obese patients undergoing general surgery (50). The present study showed that overweight class patients have a higher overall morbidity rate but not a higher mortality rate compared to normal weight and underweight patients.

### Limitations

This study has several potential limitations. First, although the FRAILSESL is a rich clinical registry, we had to adapt the NCEP-ATP III definition of MetS to the data recorded in the FRAILESEL database. In the light of this, our definition of Mets is rather deficient due to the data collection which concerns emergency conditions whose often not all data are recorded, this may have led to an uncorrected estimation of the actual incidence of Mets. Some patients with the MetS may have been “missed” because we did not include the lipid profile in our definition—and because central obesity is not always captured by a high BMI, while others could have arbitrarily included. However, our results report an incidence of MetS comparable to the literature. Furthermore, the results of this study empirically demonstrate that this syndrome, as defined here, is associated with significant morbidity. A further limitation is the design of the study with a mixed perspective and retrospective component as well as the inclusion of all diseases except malignancy; in our opinion, further studies would be useful to specifically analyze certain diseases. However, one of the primary strengths of this study is that the number of elderly patients with MetS was sufficiently large to explore the impact of this syndrome on 30-day mortality and on individual postoperative complications. Another important strength of this study is the richness of the database on which it is based. Because of the large number of clinical variables collected on the patients in the FRAILESEL database, we were able to control for many important confounders and to evaluate the impact of Mets when it is associated with both SIRS and frailty. This feature is particularly important given the fact that patients with the MetS have many comorbidities.

## Conclusions

Metabolic syndrome at admission portends a higher rate of morbidity, mortality, and longer LOS. Regarding the fact that most metabolic syndrome components can be either prevented or improved through lifestyle changes and/or pharmacological agents, a question is raised whether this can also prevent the occurrence of acute surgical conditions. Moreover, this situation demands the undertaking of a systematic review of surgical patients with MetS to articulate risks as well as the development of a care pathway to better manage these risks. Further knowledge about the nature and prevalence of complications from surgery related to MetS is needed to help formulate targeted interventional research studies aimed at improving surgical outcomes among patients with this condition. In conclusion, age per se should be not considered anymore as the main variable to estimate patient outcomes, while MetS and Frailty should have always a pivotal role.

## Data Availability

The original contributions presented in the study are included in the article/Supplementary Material, further inquiries can be directed to the corresponding author/s.

## Appendix

**List of Italian Group for Gastro-Intestinal Surgery Postoperative Surveillance (IGo-GIPS):** Agresta F, Alemanno G, Antropoli M, Argenio G, Atzeni J, Avenia N, Azzinnaro A, Baldazzi G, Barbera G, Bergamini C, Bombardini C, Borzellino G, Bozzo S, Brachini G, Buonanno GM, Canini T, Capolupo GT, Carannante F, Cardella S, Caricato M, Carrara G, Cassini D, Castriconi M, Ceccarelli G, Celi D, Ceresoli M, Chiarugi M, Cillara N, Cimino F, Cobuccio L, Cocorullo G, Colangelo E, Costa G, Crucitti A, Cucinotta E, D’Alessio R, Dalla Caneva P, de Manzoni Garberini A, De Nisco C, De Prizio M, De Sol A, Dibella A, Falcioni T, Falco N, Farina C, Finotti E, Fontana T, Francioni G, Fransvea P, Frezza B, Garbarino GM, Garulli G, Genna M, Giannessi S, Gioffrè A, Giordano A, Gozzo D, Grimaldi S, Gulotta G, Iacopini V, Iarussi T, Kurihara H, Laracca GG, Laterza E, Leonardi A, Lepre L, Luridiana G, Malagnino A, Mar G, Marini P, Mariani D, Marzaioli R, Massa G, Mecarelli V, Mercantini P, Mingoli A, Miranda G, Montuori M, Occhionorelli S, Paderno N, Palini GM, Paradies D, Paroli M, Perrone F, Petruzzelli L, Pezzolla A, Piazza D, Piazza V, Pignata G, Pinotti E, Pisanu A, Podda M, Poillucci G, Porfidia R, Rocca A, Rondelli F, Rossi G, Spagnoli A, Sulis R, Tartaglia D, Tranà C, Travaglino A, Tomaiuolo P, Valeri A, Zago M, Zanoni E.
